# Clinical Characteristics and Disease Progression in Early-Stage COVID-19 Patients in South Korea

**DOI:** 10.3390/jcm9061959

**Published:** 2020-06-23

**Authors:** Min Hyuk Choi, Hyunmin Ahn, Han Seok Ryu, Byung-Jun Kim, Joonyong Jang, Moonki Jung, Jinuoung Kim, Seok Hoon Jeong

**Affiliations:** 1 Department of Laboratory Medicine and Research Institute of Bacterial Resistance, Yonsei University College of Medicine, Seoul 06273, Korea; tcmhwd@yuhs.ac; 2 Department of Laboratory Medicine, Armed Forces Daegu Hospital, Daegu 712-906, Korea; 3 Department of Ophthalmology, Armed Forces Daegu Hospital, Daegu 712-906, Korea; overhyun@gmail.com; 4 Department of Internal Medicine, Armed Forces Daegu Hospital, Daegu 712-906, Korea; cpcforever@nate.com (H.S.R.); rla20404@naver.com (B.-J.K.); zun54@naver.com (J.J.); yessul88@naver.com (M.J.); jinote0504@gmail.com (J.K.)

**Keywords:** coronavirus disease-19 (COVID-19), severe acute respiratory syndrome coronavirus 2 (SARS-CoV-2), angiotensin II receptor blockers (ARB), ibuprofen, Korea centers for disease control and prevention (KCDC) classification

## Abstract

A rapid increase in the number of patients with coronavirus disease 19 (COVID-19) may overwhelm the available medical resources. We aimed to evaluate risk factors for disease severity in the early stages of COVID-19. The cohort comprised 293 patients with COVID-19 from 5 March 2020, to 18 March 2020. The Korea Centers for Disease Control and Prevention (KCDC) classification system was used to triage patients. The clinical course was summarized, including the impact of drugs (angiotensin II receptor blockers [ARB], ibuprofen, and dipeptidyl peptidase-4 inhibitors [DPP4i]) and the therapeutic effect of lopinavir/ritonavir. After adjusting for confounding variables, prior history of drug use, including ARB, ibuprofen, and DPP4i was not a risk factor associated with disease progression. Patients treated with lopinavir/ritonavir had significantly shorter progression-free survival than those not receiving lopinavir/ritonavir. KCDC classification I clearly distinguished the improvement/stabilization group from the progression group of COVID-19 patients (AUC 0.817; 95% CI, 0.740–0.895).

## 1. Introduction

Coronaviruses, enveloped viruses with a positive-sense single-stranded RNA genome, comprise the family Coronaviridae, order Nidovirales and are widely distributed in birds, humans, and other mammals [1]. The novel coronavirus disease (COVID-19) caused by severe acute respiratory syndrome coronavirus 2 (SARS-CoV-2) infection emerged in Wuhan, Hubei, China, on 8 December 2019 [2,3,4]. The outbreak has spread worldwide, and the number of confirmed cases is growing rapidly [5].

Most COVID-19 patients have mild symptoms, such as fever and cough [5], and have a favorable prognosis without specific treatment [4,6,7]. In severe cases, dyspnea and hypoxia may develop within one week after onset of the disease and may rapidly progress to acute respiratory distress syndrome (ARDS), acute respiratory failure, septic shock, metabolic acidosis, and coagulopathy [8].

The first case of COVID-19 in South Korea was a resident of Wuhan, China, who entered Incheon Airport on 19 January 2020 [9]. On 17 February, the 31st COVID-19 patient was confirmed to have participated in religious ceremonies in Daegu city. Subsequently, multiple COVID-19 outbreaks occurred in South Korea, including community-associated outbreaks in Daegu city and healthcare-associated outbreaks in Cheongdo, Gyeongsangbuk-do province [10].

A rapid increase in number of patients with COVID-19 can overwhelm the available medical resources, including intensive care units, negative pressure beds, and medical staff. Therefore, early assessment of risk factors for disease progression and patient prognosis is critical to ensure that patients whose disease is more likely to increase in severity can receive proper treatment in a timely manner. Thus, the Korea Centers for Disease Control and Prevention (KCDC) have established a system to triage patients in public health centers, whereby mild cases are transferred to living treatment centers, mild to moderate cases to dedicated cohort hospitals, and severe cases to tertiary university hospitals [11]. Large-scale diagnostic testing was performed to find hidden COVID-19 cases in sub-populations that had a history of contact with confirmed cases. Through these processes, South Korean patients with asymptomatic infection or early symptoms of disease were identified, and early monitoring and treatment of patients with COVID-19 were conducted.

Although many published studies have summarized the clinical features of COVID-19 patients [4,6,7,12,13,14,15], few have addressed the course of the disease in the early stages of symptom onset. Therefore, the clinical characteristics, imaging features, and treatment outcomes of COVID-19 patients before or immediately after onset of symptoms were investigated, with a particular focus on mild to moderate cases. We aimed to evaluate risk factors and KCDC classification models to predict disease progression in patients with early-stage COVID-19.

## 2. Methods

### 2.1. Study Participants

All consecutive patients with confirmed COVID-19 admitted to the Armed Forces Daegu Hospital, Daegu, South Korea, from 5 March 2020 to 18 March 2020, were enrolled in this study. According to the arrangement established by the government, the hospital was designated as a COVID-19-dedicated, 300-bed cohort hospital. The final follow-up date for this study was April 4, 2020. A total of 293 adult patients from Daegu city was admitted to the hospital during the study period. All cases were confirmed as COVID-19 using a real-time reverse transcription polymerase chain reaction (RT-PCR) (Seegene Inc., Seoul, South Korea, https://www.seegene.com) assay of nasal and oropharyngeal swabs [4]. This retrospective cohort study was approved by the Institutional Review Board of the Korean Military Medical Association (Seongnam-si, Gyeonggi-do, South Korea) (AFMC-20015-IRB-20-015).

The following clinical data were collected using electronic medical records: age at diagnosis, sex, signs and symptoms, date of symptom onset, date of hospital admission, date of discharge or transfer, Charlson comorbidity index [16], Eastern Cooperative Oncology Group (ECOG) performance status [17], Multilobular infiltration, hypo-Lymphocytosis, Bacterial coinfection, Smoking history, hyper-Tension and Age (MuLBSTA) score [18], pneumonia severity index [19], Confusion, Urea, Respiratory rate, Blood pressure plus age ≥ 65 years (CURB-65) [20], respiratory support, and treatment agents administered before and during hospitalization. We also obtained radiologic findings. Baseline non-contrast-enhanced chest computed tomography (CT) was completed for all patients to assess disease severity. To ensure the safety of medical staff by minimizing contact with patients, routine laboratory tests were not conducted in all patients but only for patients requiring clinical decisions.

### 2.2. Definitions

**Figure 1:**
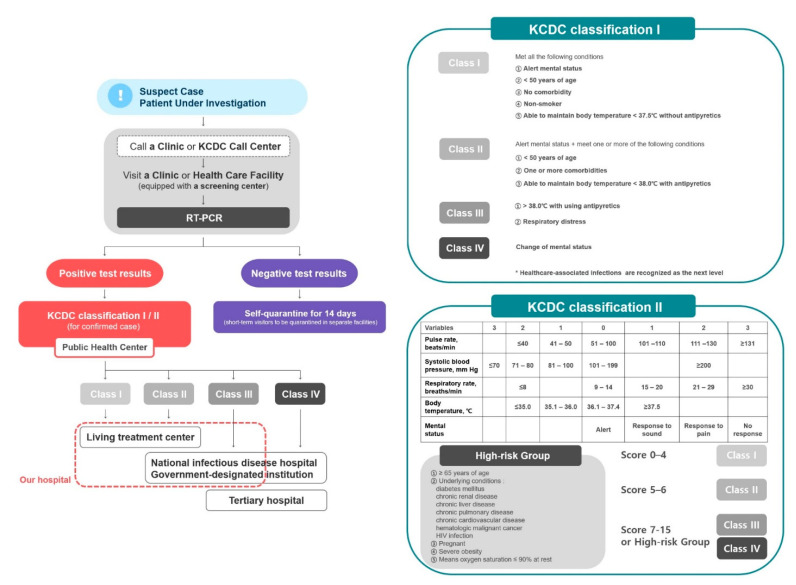
Triage algorithm and KCDC classification criteria for clinical severity of COVID-19 in South Korea. Abbreviations: KCDC, Korea Centers for Disease Control and Prevention; RT-PCR, real-time reverse transcription polymerase chain reaction Figure legend: The Living Treatment Center is a quarantine facility for mild or asymptomatic COVID-19 patients who are unable to self-isolate at home. The patients were checked for vital signs twice a day and immediately transferred to hospitals if their symptoms worsened. If their symptoms resolved, the patient was tested according to the standards for lifting the quarantine. Certain state-run facilities and accommodations are designated as Living Treatment Centers and are supplied with medical staff, medical equipment (pulse oximetry device, thermometer, blood pressure monitor, CPR kit, chest X-ray radiograph, etc.), individual relief kits (underwear, toiletries, face masks, etc.), and hygiene kits (thermometer and medical supplies).

The triage algorithm and classification criteria according to the COVID-19 response guidelines (version 7) developed by KCDC are presented in Figure 1. The criteria address the patient’s mental state, age, history of underlying comorbidities, history of smoking, respiratory symptoms, and body temperature (BT). The “KCDC Classification I” was applied if the patient’s blood pressure (BP), pulse rate (PR), and respiratory rate (RR) could not be measured, and “KCDC Classification II” was applied if these parameters could be measured; thereafter, patients with COVID-19 were classified into one of four groups (Class I to IV).

In previous studies of COVID-19 patients [8,12], “mild cases” were defined as patients who experienced mild symptoms, with no manifestations of pneumonia on chest imaging. “Moderate cases” referred to patients with uncontrolled fever despite administration of antipyretics and/or respiratory symptoms. Severe cases of COVID-19 were defined as patients with any of the following: respiratory distress, RR ≥ 30 breaths/min; mean oxygen saturation ≤ 93% at rest; arterial oxygen partial pressure/inspired oxygen fraction ≤ 300 mm Hg.

To quantify opacifications on pulmonary images, we applied the “CT score” as proposed in previous reports [21,22,23]. In brief, each pulmonary lobe was scored as 0 (none), 1 (diameter <1 cm), 2 (diameter 1 to 3 cm), 3 (diameter 3 cm to <50% of the lobe), or 4 (50% to 100% of the lobe) depending on lesion size and abnormal area. The overall score was calculated by summing all five lobar scores.

### 2.3. Outcomes

Depending on the course of the disease during hospitalization, patients were classified into either the progression or improvement/stabilization group. The progression group comprised mild or moderate cases that progressed to moderate or severe cases, while the improvement/stabilization group comprised mild cases that did not progress further. Progression-free survival (PFS) was defined as the duration of time over which patients with COVID-19 remained stable during their hospitalization.

### 2.4. Propensity Score (PS)-Matched Analyses

To adjust the outcomes of patients with COVID-19 for potential confounding factors, we conducted a PS-matched case-control study. We selected 10 variables for adjustment using univariable analyses (Table A1): age, healthcare-associated infection, ECOG performance status, asymptomatic on initial evaluation, BT at hospital admission, diastolic BP at hospital admission, PR at hospital admission, SpO_2_ at hospital admission, hypertension, and diabetes mellitus [24,25]. We then performed PS-matched analyses by attempting to match cases and control patients (1:1 matching) using the nearest-neighbor-matching method. A match occurred when the difference in the logits of the PS was <0.2 times the standard deviation (SD) of the scores.

### 2.5. Statistical Analysis

We assessed all variables using the Shapiro–Wilk test to evaluate Gaussian distributions. Descriptive statistics are presented as median and interquartile range (IQR) for continuous and categorical variables. Comparisons between groups were analyzed using the Mann–Whitney U test for continuous variables and Fisher’s exact test for categorical variables. PFS was analyzed using the Kaplan–Meier method, and differences between groups were qualified by log-rank testing. To obtain ORs and hazard ratios (HRs), univariate regressions were performed using logistic and Cox regression, respectively. All reported *p* values are two-tailed, and *p* values < 0.05 indicate statistical significance. We conducted statistical analyses using R statistical software (R Studio, Inc., https://www.r-project.org).

## 3. Results

### 3.1. Characteristics of Patients

#### 3.1.1. Before PS Matching

The demographic and clinical characteristics of the progression and improvement/stabilization groups are summarized in Table 1. The median age of the 293 patients was 29 years (IQR, 24–47 years), and 214 (73.0%) patients were male. Of the infections, 98.0% were community-associated cases. The most common symptoms at admission were productive cough (83 [28.3%]), fever (75 [25.6%]), and cough (69 [23.5%]), but more patients were asymptomatic (97 [33.1%]), and 279 patients were assigned ECOG performance status scores of zero (95.2%).

The median days from the onset of symptoms to disease confirmation was 1 day (IQR, 0–6 days), and that from onset to hospital admission was 6 days (IQR, 0–12 days). The median duration of hospitalization was 18 days (IQR, 15–20 days), and hospitalized patients had a median duration of symptoms of 7 days (IQR, 0–15 days). As of April 4, 2020, 207 (70.6%) of 293 patients had been discharged, and 2 (0.7%) patients had been transferred due to symptom aggravation. The patients’ discharge assessments were based on abatement of all symptoms, with two consecutive negative RT-PCR tests for COVID-19.

According to baseline chest CT imaging, 64 (21.8%) patients had findings consistent with bilateral pneumonia, and 56 (19.1%) patients had unilateral pneumonia. Supplementary oxygen was required in 10 patients (3.4%). One hundred patients (34.1%) were administered antibiotics empirically: the treatment regimen was quinolone (84 patients [28.7%]) or combination therapy with cefotaxime and doxycycline (14 patients [4.8%]). In addition, 30 patients (10.2%) received lopinavir/ritonavir antiviral therapy, although it was withdrawn in 21 of these 30 patients (70.0%) due to side effects such as nausea and vomiting.

Thirty-six (12.3%) cases were classified as the progression group, and the remaining 257 (87.7%) cases were classified as the improvement/stabilization group. The progression group of COVID-19 patients was significantly older than the improvement/stabilization group (49.5 vs. 27.0 years of age; *p* < 0.001). There were no statistically significant differences between sex and times from symptom onset to confirmation/ admission. The progression group included a greater proportion of cases of healthcare-associated infection than the improvement/stabilization group (*p* = 0.003). A greater proportion of patients in the progression group presented initial symptoms of fever, chest pain, dyspnea, myalgia or fatigue, chills, and diarrhea compared with patients in the improvement/stabilization group, while a greater proportion of patients in the improvement/stabilization group were asymptomatic.

Compared with the improvement/stabilization group, the progression group was more likely to have comorbidities such as hypertension (*p* = 0.003) and diabetes mellitus (*p* < 0.001). Given the greater incidence of pre-existing conditions, a greater proportion of patients in the progression group had a history of drug use, including ibuprofen (*p* = 0.044), angiotensin II receptor blockers (ARB; *p* = 0.006), calcium channel blockers (CCB; *p* = 0.047), dipeptidyl peptidase-4 inhibitors (DPP4i; *p* < 0.001), metformin (*p* < 0.001), and/or statins (*p* = 0.006).

#### 3.1.2. After PS Matching

We conducted PS matching to adjust baseline demographics and clinical variables between the progression and improvement/stabilization groups, resulting in 36 matched pairs of patients. Confounding variables were well balanced in the two groups, including all the 10 variables identified above in the Methods section (Table 2). After PS matching, prior history of drug use, including ibuprofen, ARB, DPP4i, was not statistically different between patients in the progression and improvement/stabilization groups. Similarly, the effect of these drugs on patient prognosis did not differ significantly in subgroup analysis of patients with hypertension (Table A2) or diabetes mellitus (Table A3).

### 3.2. Comparison of the Predictive Models

To confirm that KCDC classifications were suitable for initial triage of patients with COVID-19, the predictive values were compared to those of existing models using receiver operating characteristics analysis. As summarized in Table 3, all predictive values were significantly greater in the progression group than in the improvement/stabilization group (*p* < 0.001). KCDC classification I had the largest area under the curve (AUC, 0.817; 95% CI, 0.740–0.895). After incorporating the CT score measured using baseline chest CT imaging into the KCDC classification I scheme, the AUC was 0.846 (95% CI, 0.768–0.923), improving the predictive power.

### 3.3. Lopinavir/Ritonavir Treatment Outcomes

**Figure 2:**
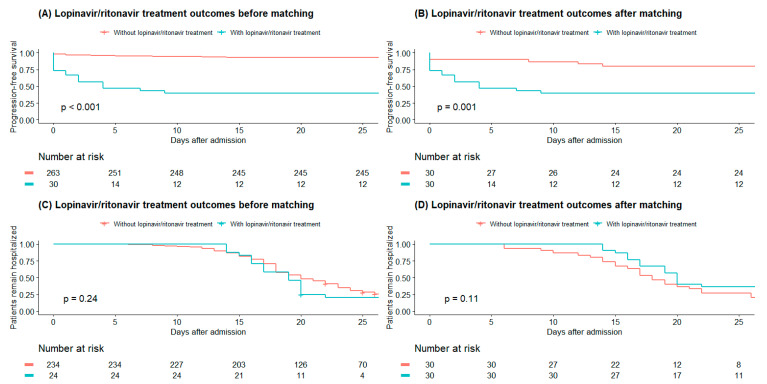
Kaplan–Meier survival analysis of progression-free survival (**A**,**B**) and proportion of patients requiring hospitalization (**C**,**D**) according to lopinavir/ritonavir treatment for patients with COVID-19 before and after propensity-score matching Figure legend: Patients treated with lopinavir/ritonavir group showed significantly lower progression-free survival than the without lopinavir/ritonavir group before and after propensity-score matching. but there was no statistical difference in discharge proportion between the two groups.

Of the 293 patients with COVID-19, 30 were treated with lopinavir/ritonavir (Table A4). Patients chosen to receive lopinavir/ritonavir treatment were more likely to be in a higher risk group than patients who did not receive lopinavir/ritonavir treatment. After adjusting for confounding variables via PS matching, there were no significant differences between the groups for any of the 10 characteristics identified in the Methods section above. However, even after matching, 18 of 30 (60.0%) patients who received lopinavir/ritonavir treatment showed disease progression, while 6 of 30 (20.0%) patients who did not receive lopinavir/ritonavir treatment experienced disease progression. Patients treated in the lopinavir/ritonavir group had significantly shorter PFS than that in the group not receiving lopinavir/ritonavir both before and after PS matching, but there was no significant difference in the proportion of discharged patients between the two groups (Figure 2 and Table A5). 

## 4. Discussions

In this cohort study, we reported the clinical characteristics of COVID-19 patients and risk factors associated with disease progression, especially those associated with early stages of the disease. We also assessed the usefulness of the KCDC classification for initial patient triage.

There are considerable differences between our study and previous studies of the course and severity of COVID-19. In previous reports addressing Chinese COVID-19 patients, most of the patients were middle-aged and elderly, presented fever and/or cough, and chest CT indicated pneumonia in most patients [4,6,7,12,13,26,27]. In our study, the median age was 29 years, 33.1% of patients were asymptomatic at hospital admission, and pneumonia was evident in chest CT scans in only 41.3% of patients. In South Korea, COVID-19 patients were identified and classified at an early stage through use of large-scale diagnostic testing in accordance with national policy, which allowed both asymptomatic and symptomatic patients to receive inpatient treatment.

The risk factors significantly associated with disease progression were older age, healthcare-associated infection, ECOG performance status, presence of initial symptoms at the time of hospital admission, higher initial PR, lower initial SpO2, hypertension, and diabetes, which were consistent with prior reports [4,6,12,21,23,28]. In contrast, asymptomatic cases at the time of hospital admission had favorable outcomes.

SARS-CoV-2 infects host cells by interacting with the angiotensin-converting enzyme (ACE) 2 receptors [29], which are expressed by epithelial cells in the lung, kidney, intestine, and blood vessels [30]. The high prevalence of ARDS and gastrointestinal symptoms such as diarrhea, nausea, and vomiting can be explained by this ACE-2-receptor-mediated mechanism in COVID-19 patients [31]. ACE inhibitors (ACEi) and ARBs that affect the renin–angiotensin–aldosterone system (RAAS) are commonly recommended for patients with hypertension [32]. In an animal model [33] and human studies [34,35], administration of ACEi and ARBs has been shown to increase the number of ACE2 receptors; ibuprofen and thiazolidinediones may also increase ACE2 expression [36]. Therefore, prior use of these drugs may be a risk factor for SARS-CoV 2 infection. Similar to the ACE2 receptor, human CD26 (also called DPP4) is also suggested as the potential binding site for COVID-19 [37]. Thus, DPP4i, which is widely used as a diabetes drug, may produce effects similar to ARBs in COVID-19 patients. However, given only small-scale clinical studies addressing ACEi/ARB use and patient outcomes in hospital settings [28] have been completed to date, the impact of these drugs on COVID-19 is controversial. Moreover, Vaduganathan et al. suggested that recombinant ACE2 protein may restore balance to the RAAS and potentially prevent organ damage, and drugs acting on ACE2 may benefit rather than harm COVID-19 patients [38]. To analyze the impact of drugs acting on the ACE2 receptor and human CD26 in COVID-19 patients, we conducted a PS matched study. Before matching, the proportion of patients reporting prior use of these drugs was significantly greater in the progression group; however, after adjusting for 10 confounding variables, including underlying comorbidities, there was no significant difference between patients with and without these medication histories. Even after comparing the sums of three (ibuprofen, ARBs, and thiazolidinediones) or four drugs (ibuprofen, ARBs, thiazolidinediones, and DPP4i), no significant differences were found between the two groups. Furthermore, in subgroup analysis of patients with hypertension and diabetes mellitus, the effect of these drugs on patient prognosis was not statistically significant. These results suggest that, in diseases such as hypertension and diabetes mellitus, the underlying pathophysiology associated with the RAAS affects the prognosis of COVID-19 patients rather than the pharmacologic effects of the drugs used to control the disease.

In the global COVID-19 pandemic, the major challenge is the lack of medical resources. We evaluated use of the KCDC classifications to triage patients with COVID-19 according to severity of the disease and to ensure they are treated at the appropriate medical institution. Our results indicated that KCDC classification I had a good AUC (0.817; 95% CI 73.98-89.46) and sensitivity, which suggested that this model is suitable for early screening of low-risk patients who are less likely to progress to severe disease. The use of the triage algorithm and KCDC classification for COVID-19 patients saves medical resources, allowing more efficient treatment and management of patients. Using the KCDC classification as a predictive model in the early stages of COVID-19 outbreaks, more medical resources could be focused on patients with more severe disease, which may have underlain the relatively low CFR in South Korea.

The CT scores (AUC > 0.7) for the COVID-19 patients in this study clearly distinguished the progression group from the improvement/stabilization group, a finding which is consistent with previous reports [21,22,23]. The use of the KCDC classification I scheme with the CT score increased the AUC and specificity of the predictive model. Therefore, we suggest that triaging patients by applying these predictive models in accordance with the medical conditions and policies of each country may help manage patients in the COVID-19 pandemic situation.

In our clinical study, which comprised mostly mild to moderate cases, patients who received lopinavir/ritonavir treatment were not likely to experience a decrease in PFS; rather, the patients’ symptoms may have been aggravated due to side effects of the antivirals. In a previous randomized controlled trial conducted in patients with severe COVID-19, there was no treatment benefit of lopinavir/ritonavir: of 95 patients receiving lopinavir/ritonavir treatment, 48 (48.4%) had gastrointestinal side effects [39], which is consistent with our data.

The retrospective and single-center nature of our study may limit wider applicability of the results. Due to the limited number of cases in the progression group, it was difficult to analyze risk factors for disease progression using multivariable-adjusted methods. Thus, hidden bias and residual confounding factors might have influenced our results. Another limitation of our study was that, to protect medical staff and minimize further spread of the disease in the hospital setting, routine laboratory tests were not conducted in all patients, and these data were not available for inclusion in the analyses. However, we tried to analyze risk factors for disease progression and treatment outcomes for COVID-19 patients while minimizing selection bias using the PS matched study.

After controlling for potential biases using PS matching analysis, drugs acting on the ACE2 receptor and human CD26 were not risk factors for disease progression. We also demonstrated that the KCDC classification I was able to distinguish the improvement/stabilization group from the progression group of COVID-19 patients, and the triage algorithm system saved medical resources, enabling efficient treatment and management of COVID-19 patients in South Korea.

## Figures and Tables

**Table 1:** Baseline characteristics of patients with COVID-19, before propensity-score matching *.

		Total, No. (%)	Progression, No. (%)	Improvement/Stabilization, No. (%)	*p*
		(*n* = 293)	(*n* = 36)	(*n* = 257)	Value
Age, median (IQR)		29 (24–47)	49.5 (34–57)	27 (23–46)	<0.001
Male sex		214 (73.0%)	24 (66.7%)	190 (73.9%)	0.472
Healthcare-associated infection		6 (2.0%)	3 (8.3%)	3 (1.2%)	0.027
ECOG performance status					<0.001
0		279 (95.2%)	28 (77.8%)	251 (97.7%)	
1		12 (4.1%)	8 (22.2%)	4 (1.6%)	
2		2 (0.7%)	0 (0.0%)	2 (0.8%)	
Time from disease confirmation to admission, median (IQR), days		5.0 (3.0–6.0)	4.0 (3.0–6.0)	5.0 (3.0–6.0)	0.712
Time from symptom onset to admission, median (IQR), days		6.0 (0.0–12.0)	8.0 (3.0–10.5)	5.0 (0.0–12.0)	0.156
Time from symptom onset to confirmation, median (IQR), days		1.0 (0.0–6.0)	3.0 (0.0–6.0)	1.0 (0.0–5.0)	0.198
Time from admission to discharge, median (IQR), days		18.0 (15.0–20.0)	18.0 (14.0–20.0)	18.0 (15.5–21.0)	0.568
Symptom duration, median (IQR), days		7.0 (0.0–15.0)	12.0 (10.0–20.0)	6.0 (0.0–15.0)	<0.001
Initial symptoms (may be multiple)					
Asymptomatic		97 (33.1%)	3 (8.3%)	94 (36.6%)	0.001
Productive cough		83 (28.3%)	15 (41.7%)	68 (26.5%)	0.089
Fever		75 (25.6%)	20 (55.6%)	55 (21.4%)	<0.001
Cough		69 (23.5%)	4 (11.1%)	65 (25.3%)	0.095
Headache		61 (20.8%)	16 (44.4%)	45 (17.5%)	<0.001
Myalgia or fatigue		60 (20.5%)	14 (38.9%)	46 (17.9%)	0.007
Chills		54 (18.4%)	19 (52.8%)	35 (13.6%)	<0.001
Sore throat		42 (14.3%)	7 (19.4%)	35 (13.6%)	0.496
Rhinorrhea		36 (12.3%)	4 (11.1%)	32 (12.5%)	>0.999
Dyspnea		22 (7.5%)	10 (27.8%)	12 (4.7%)	<0.001
Diarrhea		19 (6.5%)	7 (19.4%)	12 (4.7%)	0.003
Nausea or vomiting		9 (3.1%)	3 (8.3%)	6 (2.3%)	0.150
Chest pain		9 (3.1%)	4 (11.1%)	5 (1.9%)	0.014
Other		8 (2.7%)	1 (2.8%)	7 (2.7%)	>0.999
Initial signs (may be multiple)					
Body temperature, median (IQR), °C		36.7 (36.5–37.0)	37.3 (36.5–37.7)	36.7 (36.5–37.0)	<0.001
Systolic blood pressure, median (IQR), mm Hg		133.0 (121.0–141.0)	135.5 (121.5–146.0)	132.0 (121.0–141.0)	0.335
Diastolic blood pressure, median (IQR), mm Hg		79.0 (72.0–85.0)	82.0 (75.5–90.5)	78.0 (72.0–84.0)	0.015
Pulse rate, median (IQR), beats/min		86.0 (76.0–96.0)	90.0 (79.0–102.5)	85.0 (75.0–95.0)	0.010
Respiratory rate, median (IQR), breaths/min		16.0 (16.0–18.0)	16.0 (16.0–18.0)	16.0 (16.0–18.0)	0.105
SpO2, median (IQR), %		98.0 (98.0–99.0)	98.0 (97.0–99.0)	98.0 (98.0–99.0)	0.023
Comorbidities (may be multiple)					
Hypertension		29 (9.9%)	9 (25.0%)	20 (7.8%)	0.003
Diabetes mellitus		21 (7.2%)	10 (27.8%)	11 (4.3%)	<0.001
Allergic disease		38 (13.0%)	1 (2.8%)	37 (14.4%)	0.093
Chronic lung disease		17 (5.8%)	3 (8.3%)	14 (5.4%)	0.754
Peripheral vascular disease		13 (4.4%)	1 (2.8%)	12 (4.7%)	0.933
Malignant tumor(s)		7 (2.4%)	0 (0.0%)	7 (2.7%)	0.675
Liver disease		5 (1.7%)	1 (2.8%)	4 (1.6%)	>0.999
Congestive heart failure		6 (2.0%)	2 (5.6%)	4 (1.6%)	0.338
Cerebrovascular disease		5 (1.7%)	2 (5.6%)	3 (1.2%)	0.224
Rheumatic disease		2 (0.7%)	1 (2.8%)	1 (0.4%)	0.583
Acute myocardial infarction		1 (0.3%)	0 (0.0%)	1 (0.4%)	>0.999
Kidney disease		1 (0.3%)	0 (0.0%)	1 (0.4%)	>0.999
Prior history of drug use					
Ibuprofen		21 (7.2%)	6 (16.7%)	15 (5.8%)	0.044
Angiotensin II receptor blockers		16 (5.5%)	6 (16.7%)	10 (3.9%)	0.006
Calcium channel blocker		16 (5.5%)	5 (13.9%)	11 (4.3%)	0.047
Beta blocker		9 (3.1%)	2 (5.6%)	7 (2.7%)	0.684
Diuretics		2 (0.7%)	0 (0.0%)	2 (0.8%)	>0.999
Dipeptidyl peptidase-4 inhibitor		16 (5.5%)	8 (22.2%)	8 (3.1%)	<0.001
Metformin		12 (4.1%)	6 (16.7%)	6 (2.3%)	<0.001
Sulfonylurea		4 (1.4%)	2 (5.6%)	2 (0.8%)	0.122
Thiazolidinedione		1 (0.3%)	1 (2.8%)	0 (0.0%)	0.250
Sodium-glucose cotransporter-2 inhibitor		1 (0.3%)	0 (0.0%)	1 (0.4%)	>0.999
Gabapentinoid		1 (0.3%)	0 (0.0%)	1 (0.4%)	>0.999
Isosorbide		1 (0.3%)	1 (2.8%)	0 (0.0%)	0.250
Statin		16 (5.5%)	6 (16.7%)	10 (3.9%)	0.006
Number of drugs acting on the ACE2 receptor ^†^					<0.001
0		266 (90.8%)	26 (72.2%)	240 (93.4%)	
1		22 (7.5%)	6 (16.7%)	16 (6.2%)	
2		4 (1.4%)	3 (8.3%)	1 (0.4%)	
3		1 (0.3%)	1 (2.8%)	0 (0.0%)	
Number of drugs acting on the ACE2 and/or DDP4 ^‡^					<0.001
0		249 (85.0%)	22 (61.1%)	227 (88.3%)	
1		36 (12.3%)	9 (25.0%)	27 (10.5%)	
2		6 (2.0%)	3 (8.3%)	3 (1.2%)	
3		2 (0.7%)	2 (5.6%)	0 (0.0%)	
**Available laboratory findings, median (IQR)**					
WBC count (10^9^/L)	*n* = 26	6.3 (5.1–8.5)	6.0 (5.2–7.7)	8.5 (5.8–9.0)	0.364
Hemoglobin (g/dL)	*n* = 26	13.9 (12.6–14.9)	14.0 (12.9–14.9)	13.7 (11.8–15.1)	0.644
Platelet count (10^9^/L)	*n* = 26	198.5 (156.0–295.0)	171.0 (142.5–287.0)	255.0 (215.5–281.0)	0.140
Neutrophil count (10^9^/L)	*n* = 26	4.6 (3.0–6.3)	4.6 (3.2–5.3)	6.0 (3.4–6.8)	0.885
Lymphocyte count (10^9^/L)	*n* = 26	1.4 (1.1–1.7)	1.4 (1.0–1.7)	1.5 (1.3–2.1)	0.364
Monocyte count (10^9^/L)	*n* = 26	0.4 (0.3–0.7)	0.4 (0.3–0.7)	0.6 (0.3–0.6)	0.729
Eosinophil count (10^9^/L)	*n* = 26	0.7 (0.0–0.9)	0.3 (0.0–0.8)	1.1 (0.8–1.8)	0.004
Activated partial thromboplastin time (sec)	*n* = 21	28.8 (27.5–32.2)	30.0 (28.1–33.1)	27.5 (27.1–27.6)	0.099
Prothrombin time (sec)	*n* = 21	10.6 (10.2–11.3)	10.8 (10.4–11.4)	10.2 (9.7–10.4)	0.089
BU*N* (mg/dl)	*n* = 26	10.8 (9.7–14.6)	11.2 (10.1–14.5)	10.2 (8.6–12.8)	0.544
Serum creatinine (ng/mL)	*n* = 26	0.9 (0.7–1.0)	1.0 (0.8–1.1)	0.7 (0.6–0.9)	0.056
Total protein (g/dL)	*n* = 26	6.9 (6.5–7.6)	6.8 (6.3–7.3)	7.5 (6.8–7.8)	0.165
Albumin (g/dL)	*n* = 26	4.0 (3.7–4.2)	3.8 (3.4–4.1)	4.2 (4.0–4.4)	0.081
AST (IU/L)	*n* = 26	29.5 (23.0–69.0)	30.0 (25.5–77.0)	24.0 (21.0–38.0)	0.148
ALT (IU/L)	*n* = 26	27.5 (17.0–52.0)	30.0 (20.5–60.5)	17.0 (17.0–43.0)	0.469
Alkaline phosphatase (IU/L)	*n* = 26	75.0 (64.0–94.0)	78.0 (69.0–94.0)	67.0 (63.5–84.5)	0.563
CRP (mg/L)	*n* = 26	3.8 (0.5–6.5)	4.8 (1.5–7.7)	0.5 (0.5–2.3)	0.081

* IQR, interquartile range, ECOG, Eastern Cooperative Oncology Group performance status; SpO2, pulse oximeter oxygen saturation; ACE, angiotensin converting enzyme; DDP4, dipeptidyl peptidase 4; BUN, blood urea nitrogen; AST, aspartate aminotransferase; ALT, alanine aminotransferase; CRP, C-reactive protein. ^†^ Includes nonsteroidal anti-inflammatory drug, angiotensin II receptor blockers, and thiazolidinediones. ^‡^ Includes nonsteroidal anti-inflammatory drug, angiotensin II receptor blockers, thiazolidinediones, and dipeptidyl peptidase-4 inhibitors.

**Table 2:** Baseline characteristics of patients with COVID-19, after propensity-score matching *.

	Progression, No. (%)	Improvement/Stabilization, No. (%)	*p*
	(*n* = 36)	(*n* = 257)	Value
Age, median (IQR)	49.5 (34.0–57.0)	45.5 (27.5–54.5)	0.185
Male sex	24 (66.7%)	19 (52.8%)	0.336
Healthcare-associated infection	3 (8.3%)	2 (5.6%)	>0.999
ECOG performance status			0.109
0	28 (77.8%)	31 (86.1%)	
1	8 (22.2%)	3 (8.3%)	
2	0 (0.0%)	2 (5.6%)	
Time from disease confirmation to admission, median (IQR), days	4.0 (3.0–6.0)	5.0 (3.0–8.0)	0.222
Time from symptom onset to admission, median (IQR), days	8.0 (3.0–10.5)	10.0 (5.0–14.0)	0.194
Time from symptom onset to confirmation, median (IQR), days	3.0 (0.0–6.0)	3.0 (0.5–8.0)	0.740
Initial symptoms (may be multiple)			
Asymptomatic	3 (8.3%)	3 (8.3%)	>0.999
Productive cough	15 (41.7%)	14 (38.9%)	>0.999
Fever	20 (55.6%)	13 (36.1%)	0.156
Cough	4 (11.1%)	9 (25.0%)	0.220
Headache	16 (44.4%)	10 (27.8%)	0.220
Myalgia or fatigue	14 (38.9%)	12 (33.3%)	0.806
Chills	19 (52.8%)	9 (25.0%)	0.030
Sore throat	7 (19.4%)	10 (27.8%)	0.579
Rhinorrhea	4 (11.1%)	6 (16.7%)	0.733
Dyspnea	10 (27.8%)	4 (11.1%)	0.137
Diarrhea	7 (19.4%)	3 (8.3%)	0.307
Nausea or vomiting	3 (8.3%)	3 (8.3%)	>0.999
Chest pain	4 (11.1%)	1 (2.8%)	0.354
Initial signs (may be multiple)			
Body temperature, median (IQR), °C	37.3 (36.5–37.7)	37.1 (36.9–37.3)	0.443
Systolic blood pressure, median (IQR), mm Hg	135.5 (121.5–146.0)	136.5 (125.5–142.0)	0.897
Diastolic blood pressure, median (IQR), mm Hg	82.0 (75.5–90.5)	82.5 (74.0–89.0)	0.778
Pulse rate, median (IQR), beats/min	90.0 (79.0–102.5)	88.0 (82.0–97.5)	0.389
Respiratory rate, median (IQR), beats/min	16.0 (16.0–18.0)	16.0 (16.0–17.0)	0.108
SpO2, median (IQR), %	98.0 (97.0–99.0)	98.0 (98.0–99.0)	0.438
Comorbidities (may be multiple)			
Hypertension	9 (25.0%)	8 (22.2%)	>0.999
Diabetes mellitus	10 (27.8%)	6 (16.7%)	0.395
Allergic diseases	1 (2.8%)	3 (8.3%)	0.607
Chronic lung disease	3 (8.3%)	3 (8.3%)	>0.999
Peripheral vascular disease	1 (2.8%)	5 (13.9%)	0.201
Malignant tumor(s)	0 (0.0%)	0 (0.0%)	NA
Liver disease	1 (2.8%)	1 (2.8%)	>0.999
Cerebrovascular disease	2 (5.6%)	1 (2.8%)	>0.999
Rheumatic disease	1 (2.8%)	0 (0.0%)	>0.999
Acute myocardial infarction	0 (0.0%)	0 (0.0%)	NA
Congestive heart failure	2 (5.6%)	0 (0.0%)	0.473
Kidney disease	0 (0.0%)	0 (0.0%)	NA
Prior history of drug use			
Ibuprofen	6 (16.7%)	2 (5.6%)	0.261
Angiotensin II receptor blockers	6 (16.7%)	4 (11.1%)	0.733
Calcium channel blocker	5 (13.9%)	4 (11.1%)	>0.999
Beta blocker	2 (5.6%)	2 (5.6%)	>0.999
Diuretic	0 (0.0%)	1 (2.8%)	>0.999
Dipeptidyl peptidase-4 inhibitor	8 (22.2%)	5 (13.9%)	0.540
Metformin	6 (16.7%)	3 (8.3%)	0.476
Sulfonylurea	2 (5.6%)	1 (2.8%)	>0.999
Thiazolidinedione	1 (2.8%)	0 (0.0%)	>0.999
SGLT2 inhibitor	0 (0.0%)	0 (0.0%)	NA
Gabapentinoid	30 (83.3%)	32 (88.9%)	>0.999
Isosorbide	1 (2.8%)	0 (0.0%)	>0.999
Statin	6 (16.7%)	3 (8.3%)	0.476
Number of drugs acting on the ACE2 receptor ^†^			0.542
0	26 (72.2%)	28 (77.8%)	
1	6 (16.7%)	7 (19.4%)	
2	3 (8.3%)	1 (2.8%)	
3	1 (2.8%)	0 (0.0%)	
Number of drugs acting on the ACE2 and/or DDP4 ^‡^			0.343
0	22 (61.1%)	26 (72.2%)	
1	9 (25.0%)	9 (25.0%)	
2	3 (8.3%)	1 (2.8%)	
3	2 (5.6%)	0 (0.0%)	

* IQR, interquartile range, ECOG, Eastern Cooperative Oncology Group performance status; SpO2, Pulse Oximeter Oxygen Saturation; ACE, angiotensin converting enzyme; DDP4, dipeptidyl peptidase 4. ^†^ Includes ibuprofen, angiotensin II receptor blockers, and thiazolidinediones. ^‡^ Includes ibuprofen, angiotensin II receptor blockers, thiazolidinediones, and dipeptidyl peptidase-4 inhibitors.

**Table 3:** Predictive models for disease severity and progression of patients with COVID-19 *.

	Total, No. (%)	Progression, No. (%)	Improvement/Stabilization, No. (%)	*p*				
	(*n* = 293)	(*n* = 36)	(*n* = 257)	Value	AUC	95% CI	Sensitivity	Specificity
KCDC classification I				<0.001	0.817	(0.740–0.895)	83.30%	67.70%
Class I	180 (61.4%)	6 (16.7%)	174 (67.7%)					
Class II	91 (31.1%)	14 (38.9%)	77 (30.0%)					
Class III	22 (7.5%)	16 (44.4%)	6 (2.3%)					
KCDC classification II				<0.001	0.676	(0.590–0.762)	52.80%	82.50%
Class I	229 (78.2%)	17 (47.2%)	212 (82.5%)					
Class III	64 (21.8%)	19 (52.8%)	45 (17.5%)					
CT score, median (IQR)	0.0 (0.0–4.0)	6.2 (1.0–14.5)	0.0 (0.0–3.0)	<0.001	0.768	(0.680–0.856)	77.80%	65.00%
MuLBSTA, median (IQR)	0.0 (0.0–5.0)	5.0 (5.0–7.0)	0.0 (0.0–5.0)	<0.001	0.744	(0.662–0.825)	77.80%	63.80%
CURB65				<0.001	0.575	(0.507–0.642)	19.40%	95.30%
0	274 (93.5%)	29 (80.6%)	245 (95.3%)					
1	18 (6.1%)	6 (16.7%)	12 (4.7%)					
2	1 (0.3%)	1 (2.8%)	0 (0.0%)					
Pneumonia severity index				<0.001	0.659	(0.568–0.750)	50.00%	79.00%
Class I	221 (75.4%)	18 (50.0%)	203 (79.0%)					
Class II	53 (18.1%)	10 (27.8%)	43 (16.7%)					
Class III	17 (5.8%)	6 (16.7%)	11 (4.3%)					
Class IV	2 (0.7%)	2 (5.6%)	0 (0.0%)					
Age-adjusted Charlson comorbidity index, median (IQR)	0.0 (0.0–1.0)	1.0 (0.5–4.0)	0.0 (0.0–1.0)	<0.001	0.703	(0.610–0.795)	75.00%	56.80%

* AUC, area under the curve; CI, confidence interval; KCDC, Korea Centers for Disease Control and Prevention; CT, computed tomography, IQR, interquartile range; MuLBSTA, Multilobular infiltration, hypo-Lymphocytosis, Bacterial coinfection, Smoking history, hyper-Tension and Age; CURB65, Confusion, Urea, Respiratory rate, Blood pressure plus age ≥ 65 years.

## Appendix A

**Table A1:** Logistic regression of risk factors for COVID-19 disease progression *.

	OR	95% CI	*p*
Age	1.07	1.04–1.10	<0.001
Male sex	0.71	0.33–1.49	0.360
Healthcare-associated infection	7.70	1.49–39.71	0.015
ECOG performance status	5.68	2.07–15.56	0.001
Time from disease confirmation to admission, days	0.97	0.86–1.09	0.567
Time from symptom onset to admission, days	1.02	0.98–1.07	0.312
Time from symptom onset to confirmation, days	1.03	0.97–1.09	0.322
Time from admission to discharge, days	0.95	0.87–1.04	0.304
Symptom duration, days	1.07	1.04–1.11	0.001
Initial symptoms			
Asymptomatic	0.16	0.05–0.53	0.003
Productive cough	1.99	0.97–4.07	0.061
Fever	4.59	2.23–9.45	<0.001
Cough	0.37	0.13–1.08	0.070
Headache	3.77	1.81–7.84	<0.001
Myalgia or fatigue	2.92	1.39–6.13	0.005
Chills	7.09	3.37–14.93	<0.001
Sore throat	1.53	0.62–3.76	0.353
Rhinorrhea	0.88	0.29–2.65	0.819
Dyspnea	7.85	3.09–19.93	<0.001
Diarrhea	4.93	1.80–13.51	0.002
Nausea or vomiting	3.80	0.91–15.93	0.068
Chest pain	6.30	1.61–24.68	0.008
Initial signs			
Body temperature, °C	9.41	3.98–22.25	<0.001
Systolic blood pressure, mm Hg	1.01	0.99–1.03	0.402
Diastolic blood pressure, mm Hg	1.03	1.00–1.06	0.048
Pulse rate, beats/min	1.04	1.01–1.06	0.010
Respiratory rate, breaths/min	1.12	0.94–1.33	0.218
SpO2, %	0.71	0.55–0.91	0.007
Comorbidities			
Hypertension	3.95	1.64–9.54	0.002
Diabetes mellitus	8.60	3.34–22.17	<0.001
Allergic disease	0.17	0.02–1.28	0.085
Chronic lung disease	1.58	0.43–5.78	0.491
Peripheral vascular disease	0.58	0.07–4.63	0.610
Malignant tumors	NA		
Liver disease	1.81	0.20–16.63	0.601
Congestive heart failure	3.72	0.66–21.09	0.138
Cerebrovascular disease	4.98	0.80–30.88	0.085
Rheumatic disease	7.31	0.45–119.59	0.163
Acute myocardial infarction	NA		
Kidney disease	NA		
Prior history of drug use			
Ibuprofen	3.23	1.16–8.95	0.024
Angiotensin II receptor blocker	4.94	1.68–14.56	0.004
Calcium channel blocker	3.61	1.18–11.07	0.025
Beta blocker	2.10	0.42–10.53	0.367
Diuretic	NA		
Dipeptidyl peptidase-4 inhibitor	8.89	3.10–25.54	<0.001
Metformin	8.37	2.54–27.59	0.001
Sulfonylurea	7.50	1.02–1.55	0.048
Thiazolidinedione	NA		
Sodium–glucose cotransporter-2 inhibitors	NA		
Gabapentinoid	NA		
Isosorbide	NA		
Statin	4.94	1.68–14.56	0.004
Number of drugs acting on the ACE2 receptor ^†^	4.37	2.14–8.92	<0.001
Number of drugs acting on the ACE2 and/or DDP4 ^‡^	3.66	2.05–6.55	<0.001
KCDC classification I	8.67	4.57–16.45	<0.001
KCDC classification II	2.29	1.59–3.30	<0.001
CT score	1.22	1.15–1.31	<0.001
MuLBSTA	1.28	1.16–1.42	<0.001
CURB65	4.86	1.88–12.57	0.001
Pneumonia severity index	2.81	1.76–4.47	<0.001
Age–adjusted Charlson comorbidity index	1.73	1.36–2.20	<0.001

* OR, odds ratio; CI, confidence interval; NA, not available; ECOG, Eastern Cooperative Oncology Group performance status; SpO2, Pulse Oximeter Oxygen Saturation; KCDC, Korea Centers for Disease Control and Prevention; CT, computed tomography; MuLBSTA, Multilobular infiltration, hypo-Lymphocytosis, Bacterial coinfection, Smoking history, hyper-Tension and Age; CURB65, Confusion, Urea, Respiratory rate, Blood pressure plus age ≥ 65 years. ^†^ Includes ibuprofen, angiotensin II receptor blockers, and thiazolidinediones. ^‡^ Includes ibuprofen, angiotensin II receptor blockers, thiazolidinediones, and dipeptidyl peptidase-4 inhibitor.

**Table A2:** Subgroup analysis of the effect of prior use of drugs in COVID-19 patients with hypertension.

	Progression, No. (%)	Improvement/Stabilization, No. (%)	*p*
	(*n* = 9)	(*n* = 20)	Value
Age, median (IQR)	55 (53.0–61.0)	51.5 (48.0–55.5)	0.125
Angiotensin II receptor blocker	6 (66.7%)	10 (50.0%)	0.666
Calcium channel blocker	5 (55.6%)	11 (55.0%)	>0.999
Ibuprofen	2 (22.2%)	1 (5.0%)	0.453
Beta blocker	2 (22.2%)	2 (10.0%)	0.763
Diuretic	0 (0.0%)	2 (10.0%)	0.848

**Table A3:** Subgroup analysis of the effect of prior use of drugs in COVID-19 patients with diabetes mellitus.

	Progression, No. (%)	Improvement/Stabilization, No. (%)	*p*
	(*n* = 10)	(*n* = 11)	Value
Age, median (IQR)	52.5 (47.0–55.0)	52.0 (51.0–55.0)	0.915
Dipeptidyl peptidase-4 inhibitor	8 (80.0%)	8 (72.7%)	>0.999
Metformin	6 (60.0%)	6 (54.5%)	>0.999
Angiotensin II receptor blocker	4 (40.0%)	1 (9.1%)	0.251
Ibuprofen	1 (10.0%)	1 (9.1%)	>0.999
Thiazolidinedione	1 (10.0%)	0 (0.0%)	0.476
Sulfonylurea	2 (20.0%)	2 (18.2%)	>0.999
Sodium-glucose cotransporter-2 inhibitor	0 (0.0%)	1 (9.1%)	>0.999
Gabapentinoid	0 (0.0%)	1 (9.1%)	>0.999
Isosorbide	1 (10.0%)	0 (0.0%)	0.476

**Table A4:** Lopinavir/ritonavir treatment outcomes in COVID-19 patients before and after propensity-score matching *.

	Before Propensity-Score Matching			After Propensity-Score Matching		
	With Lopinavir/Ritonavir Treatment	Without Lopinavir/Ritonavir Treatment	*p*	With Lopinavir/Ritonavir Treatment	Without Lopinavir/Ritonavir Treatment	*p*
	No. (%) (*n* = 30)	No. (%) (*n* = 263)	Value	No. (%) (*n* = 30)	No. (%) (*n* = 30)	Value
Disease progression	18 (60.0%)	18 (6.8%)	<0.001	18 (60.0%)	6 (20.0%)	0.004
Age, median (IQR)	54 (47–59)	27 (23–46)	<0.001	54 (47–59)	51 (46–55)	0.230
Male sex	19 (63.3%)	195 (74.1%)	0.295	19 (63.3%)	9 (30.0%)	0.020
Healthcare-associated infection	4 (13.3%)	2 (0.8%)	<0.001	4 (13.3%)	2 (6.7%)	0.667
ECOG performance status			<0.001			0.041
0	19 (63.3%)	260 (98.9%)		19 (63.3%)	27 (90.0%)	
1	9 (30.0%)	3 (1.1%)		9 (30.0%)	3 (10.0%)	
2	2 (6.7%)	0 (0.0%)		2 (6.7%)	0 (0.0%)	
Time from disease confirmation to admission, median (IQR), days	3.5 (3.0–5.0)	5.0 (3.0–7.0)	0.037	3.5 (3.0–5.0)	5.0 (3.0–7.0)	0.058
Time from symptom onset to admission, median (IQR), days	6.0 (4.0–9.0)	6.0 (0.0–12.0)	0.336	6.0 (4.0–9.0)	11.0 (8.0–15.0)	0.001
Time from symptom onset to confirmation, median (IQR), days	3.0 (0.0–5.0)	1.0 (0.0–6.0)	0.283	3.0 (0.0–5.0)	5.0 (2.0–9.0)	0.032
Initial symptoms (may be multiple)						
Asymptomatic	0 (0.0%)	97 (36.9%)	<0.001	0 (0.0%)	0 (0.0%)	NA
Productive cough	12 (40.0%)	71 (27.0%)	0.199	12 (40.0%)	18 (60.0%)	0.197
Fever	18 (60.0%)	57 (21.7%)	<0.001	18 (60.0%)	16 (53.3%)	0.794
Cough	6 (20.0%)	63 (24.0%)	0.798	6 (20.0%)	8 (26.7%)	0.760
Headache	17 (56.7%)	44 (16.7%)	<0.001	17 (56.7%)	9 (30.0%)	0.068
Myalgia or fatigue	13 (43.3%)	47 (17.9%)	0.002	13 (43.3%)	7 (23.3%)	0.171
Chills	16 (53.3%)	38 (14.4%)	<0.001	16 (53.3%)	10 (33.3%)	0.193
Sore throat	9 (30.0%)	33 (12.5%)	0.021	9 (30.0%)	7 (23.3%)	0.770
Rhinorrhea	2 (6.7%)	34 (12.9%)	0.486	2 (6.7%)	2 (6.7%)	>0.999
Dyspnea	11 (36.7%)	11 (4.2%)	<0.001	11 (36.7%)	4 (13.3%)	0.074
Diarrhea	8 (26.7%)	11 (4.2%)	<0.001	8 (26.7%)	1 (3.3%)	0.030
Nausea or vomiting	4 (13.3%)	5 (1.9%)	0.004	4 (13.3%)	1 (3.3%)	0.350
Chest pain	3 (10.0%)	6 (2.3%)	0.078	3 (10.0%)	2 (6.7%)	>0.999
Initial signs (may be multiple)						
Body temperature, median (IQR), °C	37.2 (36.5–37.5)	36.7 (36.5–37.0)	0.010	37.2 (36.5–37.5)	37.0 (36.6–37.3)	0.784
Systolic blood pressure, median (IQR), mm Hg	133.5 (115.0–140.0)	133.0 (122.0–141.0)	0.392	133.5 (115.0–140.0)	133.5 (123.0–142.0)	0.395
Diastolic blood pressure, median (IQR), mm Hg	81.0 (74.0–86.0)	78.0 (72.0–85.0)	0.379	81.0 (74.0–86.0)	75.5 (73.0–82.0)	0.411
Pulse rate, median (IQR), beats/min	87.0 (77.0–98.0)	86.0 (75.5–95.5)	0.340	87.0 (77.0–98.0)	89.0 (80.0–100.0)	0.700
Respiratory rate, median (IQR), beats/min	17.0 (16.0–18.0)	16.0 (16.0–18.0)	0.098	17.0 (16.0–18.0)	16.0 (16.0–20.0)	0.666
SpO2, median (IQR), %	98.0 (97.0–99.0)	98.0 (98.0–99.0)	0.031	98.0 (97.0–99.0)	98.0 (97.0–99.0)	0.284
Comorbidities (may be multiple)			0.000			0.000
Hypertension	9 (30.0%)	20 (7.6%)	<0.001	9 (30.0%)	7 (23.3%)	0.770
Diabetes mellitus	8 (26.7%)	13 (4.9%)	<0.001	8 (26.7%)	5 (16.7%)	0.531
Allergic disease	0 (0.0%)	38 (14.4%)	0.052	0 (0.0%)	6 (20.0%)	0.031
Chronic lung disease	2 (6.7%)	15 (5.7%)	>0.999	2 (6.7%)	4 (13.3%)	0.667
Peripheral vascular disease	1 (3.3%)	12 (4.6%)	>0.999	1 (3.3%)	5 (16.7%)	0.197
Malignant tumors	2 (6.7%)	5 (1.9%)	0.323	2 (6.7%)	2 (6.7%)	>0.999
Liver disease	0 (0.0%)	5 (1.9%)	0.986	0 (0.0%)	0 (0.0%)	NA
Congestive heart failure	3 (10.0%)	3 (1.1%)	0.010	3 (10.0%)	0 (0.0%)	0.236
Cerebrovascular disease	1 (3.3%)	4 (1.5%)	>0.999	1 (3.3%)	2 (6.7%)	>0.999
Rheumatic disease	1 (3.3%)	1 (0.4%)	0.490	1 (3.3%)	0 (0.0%)	>0.999
Acute myocardial infarction	1 (3.3%)	0 (0.0%)	0.189	1 (3.3%)	0 (0.0%)	>0.999
Kidney disease	0 (0.0%)	1 (0.4%)	>0.999	0 (0.0%)	0 (0.0%)	NA
Prior history of drug use						
Ibuprofen	6 (20.0%)	15 (5.7%)	0.012	6 (20.0%)	6 (20.0%)	>0.999
Angiotensin II receptor blocker	5 (16.7%)	11 (4.2%)	0.015	5 (16.7%)	4 (13.3%)	>0.999
Calcium channel blocker	6 (20.0%)	10 (3.8%)	0.001	6 (20.0%)	2 (6.7%)	0.255
Beta blocker	3 (10.0%)	6 (2.3%)	0.078	3 (10.0%)	1 (3.3%)	0.605
Diuretic	0 (0.0%)	2 (0.8%)	>0.999	0 (0.0%)	1 (3.3%)	>0.999
Dipeptidyl peptidase-4 inhibitor	7 (23.3%)	9 (3.4%)	<0.001	7 (23.3%)	5 (16.7%)	0.747
Metformin	6 (20.0%)	6 (2.3%)	<0.001	6 (20.0%)	3 (10.0%)	0.470
Sulfonylurea	2 (6.7%)	2 (0.8%)	0.070	2 (6.7%)	1 (3.3%)	>0.999
Thiazolidinediones	0 (0.0%)	1 (0.4%)	>0.999	0 (0.0%)	0 (0.0%)	NA
Sodium-glucose cotransporter-2 inhibitor	0 (0.0%)	1 (0.4%)	>0.999	0 (0.0%)	0 (0.0%)	NA
Gabapentinoid	0 (0.0%)	1 (0.4%)	>0.999	0 (0.0%)	0 (0.0%)	NA
Isosorbide	1 (3.3%)	0 (0.0%)	0.189	1 (3.3%)	0 (0.0%)	>0.999
Statin	7 (23.3%)	9 (3.4%)	<0.001	7 (23.3%)	4 (13.3%)	0.505
Number of drugs acting on the ACE2 receptor ^†^			<0.001			0.577
0	21 (70.0%)	245 (93.2%)		21 (70.0%)	22 (73.3%)	
1	6 (20.0%)	16 (6.1%)		6 (20.0%)	7 (23.3%)	
2	3 (10.0%)	1 (0.4%)		3 (10.0%)	1 (3.3%)	
3	0 (0.0%)	1 (0.4%)		0 (0.0%)	0 (0.0%)	
Number of drugs acting on the ACE2 receptor and/or DDP4 ^‡^			<0.001			0.706
0	17 (56.7%)	232 (88.2%)		17 (56.7%)	17 (56.7%)	
1	9 (30.0%)	27 (10.3%)		9 (30.0%)	11 (36.7%)	
2	3 (10.0%)	3 (1.1%)		3 (10.0%)	2 (6.7%)	
3	1 (3.3%)	1 (0.4%)		1 (3.3%)	0 (0.0%)	

* ECOG, Eastern Cooperative Oncology Group performance status; SpO2, Pulse Oximeter Oxygen Saturation; ACE, angiotensin converting enzyme; DDP4, dipeptidyl peptidase 4. ^†^ Includes ibuprofen, angiotensin II receptor blockers, and thiazolidinediones. ^‡^ Includes ibuprofen, angiotensin II receptor blockers, thiazolidinediones, and dipeptidyl peptidase-4 inhibitor.

**Table A5:** Cox regression of risk factors for progression-free survival of COVID-19 patients *.

	HR	95% CI	*p*
Age	1.061	1.04–1.09	<0.001
Male ex	0.7284	0.36–1.46	0.370
Healthcare-acquired infection	6.214	1.90–20.30	0.002
ECOG performance status	3.373	1.89–6.02	<0.001
Time from disease confirmation to admission, days	0.9714	0.87–1.09	0.607
Time from symptom onset to admission, days	1.02	0.98–1.06	0.330
Time from symptom onset to confirmation, days	1.025	0.97–1.08	0.344
Initial symptoms			
Asymptomatic	0.169	0.05–0.55	0.003
Productive cough	1.85	0.95–3.59	0.069
Fever	4.139	2.14–7.99	<0.001
Cough	0.3916	0.14–1.11	0.077
Headache	3.44	1.78–6.64	<0.001
Myalgia or fatigue	2.704	1.38–5.29	0.004
Chill	5.983	3.11–11.52	<0.001
Sore throat	1.467	0.64–3.35	0.363
Rhinorrhea	0.8683	0.31–2.46	0.790
Dyspnea	6.065	2.92–12.59	<0.001
Diarrhea	4.33	1.90–9.90	0.001
Nausea or vomiting	3.423	1.05–11.17	0.041
Chest pain	5.574	1.97–15.79	0.001
Initial signs			
Body temperature, °C	4.033	2.74–5.94	<0.001
Systolic blood pressure, mm Hg	1.01	0.99–1.03	0.402
Diastolic blood pressure, mm Hg	1.027	1.00–1.06	0.046
Pulse rate, beats/min	1.035	1.01–1.06	0.007
Respiratory rate, beats/min	1.106	0.94–1.30	0.217
SpO2, %	0.7119	0.56–0.90	0.005
Comorbidities			
Hypertension	3.56	1.67–7.58	0.001
Diabetes mellitus	6.59	3.17–13.69	<0.001
Allergic disease	0.1782	0.02–1.30	0.089
Chronic lung disease	1.473	0.45–4.80	0.521
Peripheral vascular disease	0.6158	0.084–4.50	0.633
Cerebrovascular disease	4.71	1.13–19.62	0.033
Rheumatic disease	4.95	0.68–36.20	0.115
Congestive heart failure	3.239	0.78–13.49	0.107
Prior history of drug use			
Ibuprofen	2.722	1.13–6.54	0.025
Angiotensin II receptor blocker	4.261	1.77–10.25	0.001
Calcium channel blocker	3.251	1.26–8.37	0.015
Beta blocker	2.056	0.49–8.56	0.322
Dipeptidyl peptidase-4 inhibitor	6.878	3.13–15.12	<0.001
Metformin	6.196	2.58–14.91	<0.001
Sulfonylurea	5.775	1.39–24.08	0.016
Thiazolidinedione	42.2	5.41–329.10	<0.001
Isosorbide	17.69	2.35–132.90	0.005
Statin	4.46	1.86–10.73	0.001
KCDC classification I	6.172	3.87–9.85	<0.001
KCDC classification II	2.137	1.54–2.97	<0.001
CT score	1.191	1.13–1.25	<0.001
MuLBSTA	1.253	1.15–1.37	<0.001
CURB65	4.142	1.97–8.73	<0.001
Pneumonia severity index	2.539	1.74–3.70	<0.001
Age-adjusted Charlson comorbidity index	1.608	1.33–1.94	<0.001

* HR, hazard ratio; CI, confidence interval; NA; ECOG, Eastern Cooperative Oncology Group performance status; SpO2, Pulse Oximeter Oxygen Saturation; KCDC, Korea Centers for Disease Control and Prevention; CT, computed tomography; MuLBSTA, Multilobular infiltration, hypo-Lymphocytosis, Bacterial coinfection, Smoking history, hyper-Tension and Age; CURB65, Confusion, Urea, Respiratory rate, Blood pressure plus age ≥ 65 year.
